# Beta-Arrestin 1 Mediates Liver Thyrotropin Regulation of Cholesterol Conversion Metabolism via the Akt-Dependent Pathway

**DOI:** 10.1155/2018/4371396

**Published:** 2018-05-02

**Authors:** Shaona Niu, Hui Li, Wenbin Chen, Jiajun Zhao, Ling Gao, Tao Bo

**Affiliations:** ^1^Shandong Key Laboratory of Endocrinology and Lipid Metabolism, Institute of Endocrinology and Metabolism, Shandong Academy of Clinical Medicine, Jinan, Shandong 250021, China; ^2^Department of Endocrinology, Lin Yi People's Hospital Affiliated to Shandong University, Linyi, Shandong 276003, China; ^3^Medical College, Shandong University, Jinan, Shandong 250012, China; ^4^Scientific Center, Shandong Provincial Hospital Affiliated to Shandong University, Jinan, Shandong 250021, China; ^5^Department of Endocrinology and Metabolism, Shandong Provincial Hospital Affiliated to Shandong University, Jinan, Shandong 250021, China

## Abstract

After activation, G protein-coupled receptors (GPCRs) are desensitized by *β*-arrestins (ARRBs). Moreover, ARRBs can initiate a second wave of signaling independent of G proteins. Thyroid-stimulating hormone receptor (TSHR) is one of the GPCR members. In our previous study, TSHR was identified in the liver; the major role of TSHR in cholesterol metabolism was illustrated, as TSH could regulate hepatic cholesterol metabolism via cAMP/PKA/CREB/HMGCR and SREBP2/HNF4*α*/CYP7A1 pathways. It has been reported that ARRB2 predominates over ARRB1 in TSHR internalization. However, the significance of ARRBs in TSH-initiated cholesterol metabolism has not been illustrated. In our study, the effects of ARRBs on TSH-regulated cholesterol metabolism are investigated. ARRB1/2 was genetically inactivated in C57BL/6 mice and HepG2 cell line, respectively. Cholesterol levels in arrestin-knockout mice and arrestin-knockdown cells were measured. Molecules participating in cholesterol metabolism were analyzed. It turned out that deficiencies in ARRB1 led to decreased cholesterol levels and decreased TSH-stimulated AKT phosphorylation. Subsequently, the inhibitory effect on CYP7A1 by SREBP2 was reduced due to lowered mature SREBP2 level. Other than the failures of TSH in ARRB-knockdown cells, the AKT activator SC79 could enhance AKT phosphorylation and mature SREBP2 level. Our results demonstrate that ARRBs, especially ARRB1, are involved in TSH-regulated cholesterol metabolism through the AKT pathway.

## 1. Introduction

G protein-coupled receptors (GPCRs) are known as the largest superfamily of signal transduction and transmission molecules [1]. Since GPCRs are involved in many diseases, they have long been considered targets for drug therapies [2]. GPCRs are seven-transmembrane receptors (7TMRs) that undergo a conformational variation that leads to coupling and activation of heterotrimeric G proteins. Activated G proteins then promote the generation of second messengers, including cyclic adenosine monophosphate (cAMP), calcium, or phosphoinositides [3].

After activation, GPCRs are desensitized and internalized by *β*-arrestins (ARRBs). ARRBs can inhibit GPCR signaling by binding to GPCRs and uncoupling the receptors from their G protein subunits. Additionally, it has been reported that ARRBs can initiate a second wave of signal transmission independent of G proteins through signaling pathways mediated by molecules such as protein kinase B (AKT) and ERK; in these pathways, ARRBs act as multifunctional adaptors [4–7]. This so-called biased signal transformation has been shown to be involved in several metabolic processes. It has been reported that *β*-arrestin 1 (ARRB1), not *β*-arrestin 2 (ARRB2), enhances hepatocellular carcinogenesis by inflammation-mediated AKT signaling [8]. Activated ARRB1 can facilitate AKT-mediated p53 degradation by acting as a molecular scaffold, and this process plays important roles in stress response pathways that regulate *β*2-adrenoreceptor-mediated DNA damage [9].

Based on their different binding affinities to ARRB isoforms, GPCRs can be divided into two classes. Class A receptors show a higher affinity to ARRB2 than to ARRB1, and class B receptors show similar affinity to both ARRB1 and ARRB2 [10]. Thyroid-stimulating hormone receptor (TSHR) was one of the first reported members of the class A receptors, as ARRB2 plays a predominant role over ARRB1 in TSHR internalization [11]. TSHR is one of the GPCR members that mediate the function of thyrotropin (TSH) through highly specific interactions [12]. Several studies have demonstrated that TSHR was detected in many cells and tissues other than those in the thyroid, such as in lymphocytes, adipocytes, retroocular fibroblasts, erythrocytes, osteocytes, neuronal cells, and astrocytes [13–18]. The specific effects of TSHR in these nonthyroid tissues have not been clearly illustrated. In our previous study, hepatic TSHR was detected, with the same mRNA sequence as that in the thyroid. TSHR protein was also expressed and was mainly located in the hepatocyte cell membrane [19]. Moreover, TSH was shown to initiate the cyclic adenosine monophosphate/protein kinase A/cyclic adenosine monophosphate-responsive element binding (cAMP/PKA/CREB) protein signaling system, which is mediated by liver TSHR. TSH was shown to subsequently elevate the expression level of a rate-limiting enzyme in cholesterol synthesis, 3-hydroxy-3-methyl-glutaryl coenzyme A reductase (HMGCR) [20]. However, TSH-mediated cholesterol conversion was not dependent on PKA/cAMP, but rather on PI3K/AKT and sterol-regulatory element-binding protein-2 (SREBP2), which is a cholesterogenic transcription factor involved in the regulation of cholesterol metabolism-related genes [21, 22]. SREBP2 regulates the transcription level of cholesterol 7-alpha hydroxylase (CYP7A1), which is a key component involved in the bile acid (BA) synthesis pathway [23]. However, the particular metabolic significance of liver ARRBs in TSH-initiated cholesterol metabolism pathways has not been illustrated yet. On the one hand, ARRB may serve as a TSHR negative regulator, participating in the reported PKA/cAMP pathway. On the other hand, considering the known close relationship between AKT and SREBP2, the ARRB-dependent signaling pathway may be included in cholesterol regulation. In the present work, the cholesterol metabolism molecules downstream of the traditional GPCR-dependent pathway and the biased signaling pathway of ARRBs were analyzed in *arrb1/2*-knockout C57BL/6 mice and an *ARRB1/2*-knockdown HepG2 cell line. The detailed functions of ARRBs in TSH-regulated cholesterol conversion in the liver are discussed.

## 2. Materials and Methods

### 2.1. Animal Experiments

Genetically *arrb1^−/−^* (GenBank: NC_000073) and *arrb2^−/−^* (GenBank: NC_000077) mice (C57BL/6J) were kindly provided by Professor Jinpeng Sun, Medical College of Shandong University. The wild-type mice and genetically manipulated mice were housed at 23°C under a 12-hour light-dark cycle and in a humidity-controlled (60%) environment.

To generate a subclinical hypothyroidism (SCH) mouse model, male C57BL/6 mice (8 weeks old) were given methimazole (MMI, 0.04 mg/kg·d.) in drinking water to inhibit thyroid hormone (TH) synthesis. Mice were weighed each week, and the MMI doses were adjusted according to the body weight. After MMI was administered for 12 weeks, the mice were fasted for 6 h and then euthanized using sodium pentobarbital. Serum samples were collected immediately prior to sacrificing the mice and were tested for fT4 and TSH levels. Livers were excised, washed in PBS, and stored in liquid nitrogen for further analysis. Total mRNA was extracted using RNAiso Plus (Takara, number 9108). Total protein was extracted according to standard protocols. Briefly, tissues were ground into powder in liquid nitrogen and then lysed in RIPA buffer (Solarbio, R0010) to obtain total protein. The protein concentration was measured using the BCA method (Applygen Technologies Inc., P1511).

### 2.2. Cell Culture

HepG2 cells were cultured in Dulbecco's modified Eagle's medium (DMEM, Gibco, Life Technologies Corporation, USA) supplemented with 10% fetal bovine serum (Gibco, Life Technologies Corporation, USA) and 100 U/mL penicillin-streptomycin. The cells were starved overnight when they reached 70–80% confluence, 4 *μ*M bovine TSH (T8931, Sigma-Aldrich, USA) was added, and stimulation was conducted for a time course of 1 hour or 24 hours in serum-free DMEM. Total protein was extracted according to standard protocols. SC79 (Abcam, ab146428) was used as an AKT activator.

### 2.3. Genetic Manipulation of ARRBs

The CRISPR/Cas9 system was used for cellular genetic manipulation to generate a genetically stable gene-knockdown cell line. Briefly, HepG2 cells were cultured to 70–80% confluence and then transfected with the lentiviral vector Lenti-Cas9-EGFP. Puromycin was used to select a stable Cas9 expression strain. HepG2 cells with stable expression of Cas9 (described as HepG2-Cas9 in our research) were selected, subcultured with puromycin (10 *μ*M, A1113803, Thermo Fisher Scientific), and subsequently used for target gene knockdown. sgRNAs of human *ARRB1* (GenBank: NC_000011) and *ARRB2* (GenBank: NC_000017) were transfected into HepG2-Cas9 to obtain *ARRB1/2* gene-knockdown cell lines. The sequences of sgRNAs are listed as follows: *ARRB1*, 5′-TTCTTGTCCTCGGGGGCCGG-3′, and *ARRB2*, 5′-CCTGTTCATCGCCACCTACC-3′. sgRNA sequences were designed by http://crispr.mit.edu/. For each target gene, 3 sgRNAs were designed and transfected into HepG2-Cas9 cells; the knockdown efficiencies were analyzed by Western blot to screen the most effective sequence.

### 2.4. GloSensor™ cAMP Assay

The cAMP assay was performed by the GloSensor assay (Promega), according to the manufacturer's instruction. Briefly, HepG2 cells were plated on 96-well plates at a density of 10000 cells per well. The pGloSensor™-22F cAMP plasmid was transfected into cells using Lipofectamine 3000 (Invitrogen). The cells were maintained in culture medium for 24 hours; then, the medium was changed with 100 *μ*L of CO_2_-independent medium with 2% *v*/*v* GloSensor cAMP Reagent (E1171, Promega) and 10% FBS for two hours. The cAMP signal was initiated by adding TSH, with forskolin (10 *μ*M, according to the manufacturer) and PBS as positive and negative controls, respectively. GloSensor luciferase activities were measured on a microplate luminometer (Berthold Centro XS^3^ LB960).

### 2.5. Cholesterol Measurements

Free and total cholesterol levels in tissues and cells were measured using cholesterol assay kits applied by Applygen Technologies Inc. (E1015, E1016). All procedures were performed according to standard protocols. Briefly, approximately 100 mg of tissue samples or 10^7^ cells were harvested and lysed in lysis buffer, and the supernatants were divided into two parts, one for cholesterol measurements and the other for analysis of protein concentration. Calibration curves were determined by measuring the optical density (OD) of a series dilution of standards at the wavelength of 550 nm. The final cholesterol levels were standardized by protein concentration and were expressed as mmol cholesterol per gram of protein. Sample protein concentrations were measured using the BCA assay (Applygen Technologies Inc., P1511).

### 2.6. Molecular Analysis of Cholesterol Metabolism-Related Proteins

After animals were executed, tissue samples were extracted and immediately stored in liquid nitrogen. When in use, tissues were ground in liquid nitrogen, and total mRNA was extracted by the TRIzol (Invitrogen, 15596026) assay, according to the manufacturers. Transcription levels of target genes were analyzed by measuring mRNA level using real-time quantitative PCR (qRT-PCR). For immunoblotting analysis, tissue protein and cell protein were extracted by the RIPA assay (Solarbio, R0010) according to the standard protocol of the manufacturers. Relative primers and antibodies are listed as follows: mouse *arrb1* primers—forward, 5′-CGGATGCTTTCTCGTCTC-3′, and reverse, 5′-ACCCATCATCATTGTGCC-3′, and mouse *arrb2* primers—forward, 5′-AGGAACTCTGTGCGGCTTAT-3′, and reverse, 5′-CCACGGGACACTTGTACTGC-3′. Anti-HMGCR (Abcam, ab174830), anti-CYP7A1 (Abcam, ab65596), anti-phosphorylated AKT (CST, #4060, #4056), anti-*β*-actin (Zhongshan Jinqiao, Beijing, TA336770), and anti-SREBP2 antibodies were produced by ChinaPeptides, Suzhou, China.

### 2.7. Graphics and Statistical Analysis

The data were analyzed using SPSS 17.0 and expressed as the mean ± SE. Error bars represent the standard error (SEM). The density of immunoblotting bands was compared on the densitometric scan by ImageJ (National Institutes of Health, Bethesda, Maryland), quantified by Origin 9.0, and set as grayscale analysis. The statistical significance was assessed by *t*-tests for differences between two experimental groups and by one-way ANOVA for multiple groups. *p* < 0.05 was considered statistically significant. Graphics were generated using Origin 9.0 and assembled in Adobe Illustrator CS5.

## 3. Results

### 3.1. TSH Influences Transcriptional and Translational Levels of ARRBs

**Figure 1:**
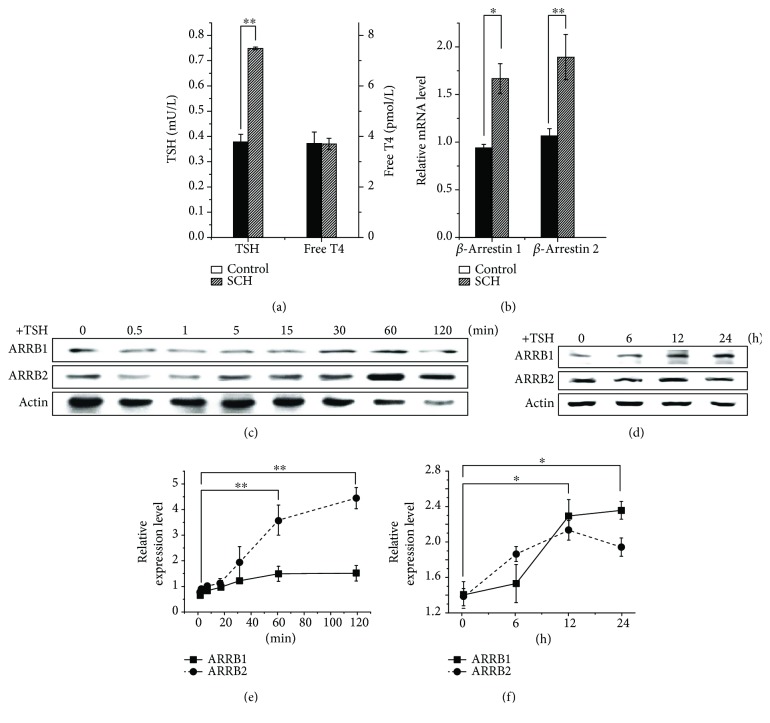
TSH influences transcriptional and translational levels of *β*-arrestins. Liver tissues of SCH mice and wild-type counterparts, which have the same serum free T4 level but different TSH level, were extracted, and mRNA transcriptional analysis of *β*-arrestins was performed through qRT-PCR. Protein samples of HepG2 cells treated with 4 *μ*M TSH were extracted in a time course and analyzed by Western blot. (a) TSH and free T4 level of SCH mice and control individuals. (b) Relative mRNA level of *β*-arrestins in the liver of SCH mice and control individuals. (c, d) Immunoblotting analysis of *β*-arrestin expression in HepG2 cells under 4 *μ*M TSH stimulation in 2 and 24 hours. (e, f) Grayscale analysis of *β*-arrestin expression in HepG2 cells under 4 *μ*M TSH stimulation for 2 and 24 hours. All experiments were repeated 3 times with similar results. The differences between groups were compared by using nonparametric tests. ^∗^*p* < 0.05 versus control group. ^∗∗^*p* < 0.01 versus control group. Error bars are calculated as a standard error (SEM).

Serum and liver tissues of SCH and wild-type controls were harvested. Each group contained three animals. The serum TSH and free T4 were measured, and the liver mRNA was extracted for the analysis of *ARRB* transcriptional level by qRT-PCR. The results are shown in Figure 1. The serum TSH levels in SCH mice were significantly higher than those in their wild-type counterparts, but the levels of free T4 were similar (Figure 1(a)). As a result, compared to wild type, ARRB2 in the livers of SCH mice showed an increase in mRNA level, with a statistical significance (*p* < 0.01, Figure 1(b)). ARRB1 showed less differences than ARRB2, with a statistical significance (*p* < 0.05). These results indicated that ARRB2 varied with TSH. In HepG2 cell lines, immunoblotting analysis showed that, although 4 *μ*M TSH stimulation led to a detectable increase in expression levels of ARRBs over 24 hours (Figures 1(d) and 1(f)), a dramatic increase in ARRB2 was observed over 60–120 min, whereas ARRB1 increased slightly within 120 min, with no statistical significance (Figures 1(c) and 1(e)). These results may suggest that, as a member of the GPCR family, activated liver TSHR may need ARRBs as negative regulatory factors and ARRB2 may play more important roles in TSHR internalization.

### 3.2. ARRB Deficiencies Lead to Decreased Cholesterol Levels

**Figure 2:**
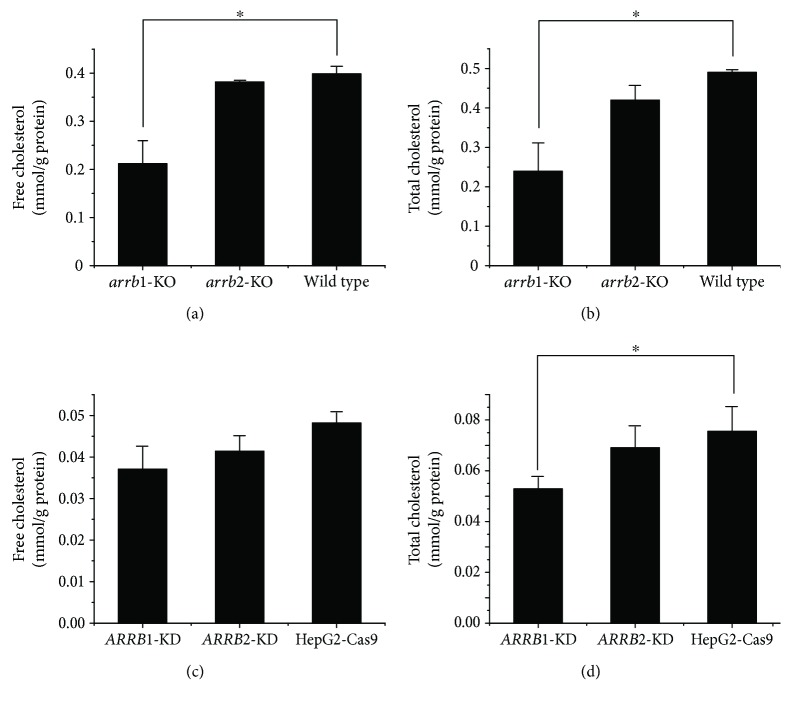
*β*-Arrestin deficiencies lead to decreased cholesterol levels. Free and total cholesterol levels of liver tissues of *arrb1/2*-knockout (KO) mice (a, b) and *ARRB1/2*-knockdown (KD) HepG2 cells (c, d). Cholesterol levels were standardized by protein concentration and were expressed as mmol cholesterol per gram of protein. The differences between groups were compared by using nonparametric tests. ^∗^*p* < 0.05 versus control group. Error bars are calculated as a standard error (SEM).

Cholesterol levels were analyzed in the liver tissues of *arrb1*^−/−^ mice and *arrb2*^−/−^ mice. Free and total cholesterol levels in the liver tissues of *arrb1*^−/−^ mice were lower than those of their wild-type counterparts, whereas *arrb2*^−/−^ mice did not show obvious differences (Figures 2(a) and 2(b)). We next measured the cholesterol level in HepG2-Cas9 cells transfected with *ARRB1/2* sgRNA (termed *ARRB1/2*-KD in our research), in which the expression levels of *ARRB1/2* were dramatically reduced. Cells were harvested and cholesterol levels were analyzed following protein concentration normalization, and the results are shown in Figures 2(c) and 2(d). Compared to the control cells, total cholesterol level of the *ARRB1*-KD cells showed a dramatic decrease, whereas a slight decrease was observed in the *ARRB2*-KD cells, with no significant differences from the control. These results demonstrated that ARRB1 may play more important roles than ARRB2 in hepatic cholesterol metabolism.

### 3.3. Functions of ARRBs in TSH-Stimulated Cholesterol Metabolism

**Figure 3:**
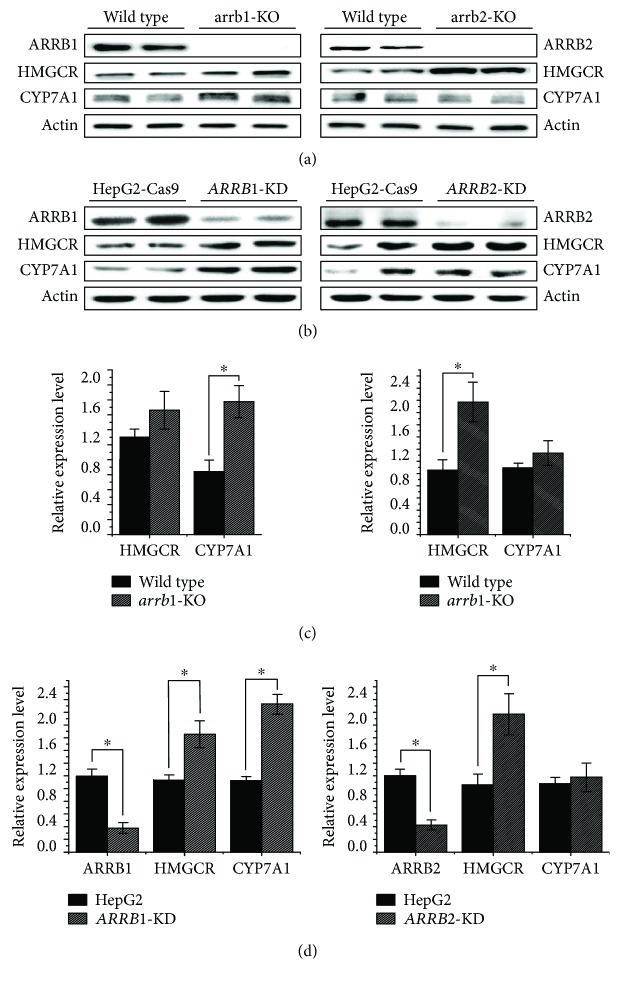
Immunoblotting analysis of cholesterol metabolism-related proteins in *β*-arrestin-intervened animals and cells. (a) *arrb1/2*-KO mice and wild-type littermates were cultivated to 12 weeks, mice were sacrificed, and protein samples of liver tissues were extracted for Western blot using the indicated antibodies. (b) HepG2-Cas9 cells and *ARRB1/2*-knockdown HepG2-Cas9 cells were cultured to 70–80% confluence, and protein samples were extracted and analyzed by Western blot. All experiments were repeated 3 times with similar results. (c) Grayscale analysis of relative expression level of ARRBs, HMGCR, and CYP7A1 of *arrb1/2*-KO mice and wild-type littermates. (d) Grayscale analysis of relative expression level of ARRBs, HMGCR, and CYP7A1 of HepG2-Cas9 cells and *ARRB1/2*-knockdown HepG2-Cas9 cells. ^∗^*p* < 0.05. Error bars are calculated as a standard error (SEM).

To illustrate the principal roles of ARRBs in the cholesterol metabolism pathway in detail, components involved in the cholesterol synthesis and conversion pathways were analyzed by Western blot in both genetically manipulated animals and cell lines. The results are shown in Figure 3. In the livers of *arrb1/2*-knockout mice, the level of *HMGCR* was increased on account of ARRB deficiency, which indicated that cholesterol synthesis had been upregulated. For the cholesterol conversion process, CYP7A1 showed increased expression in response to *arrb1* deficiency, which illustrated that the level of bile acids transformed by cholesterol was increased (Figures 3(a) and 3(c)). As we predicted, HepG2-Cas9 cells transfected with *ARRB1/2* sgRNA showed a dramatic decrease in the protein levels of ARRB1 and ARRB2 (Figures 3(b) and 3(d)). As a result, HMGCR and CYP7A1 increased, showing a similar tendency to that in *arrb1/2*-KO animals.

**Figure 4:**
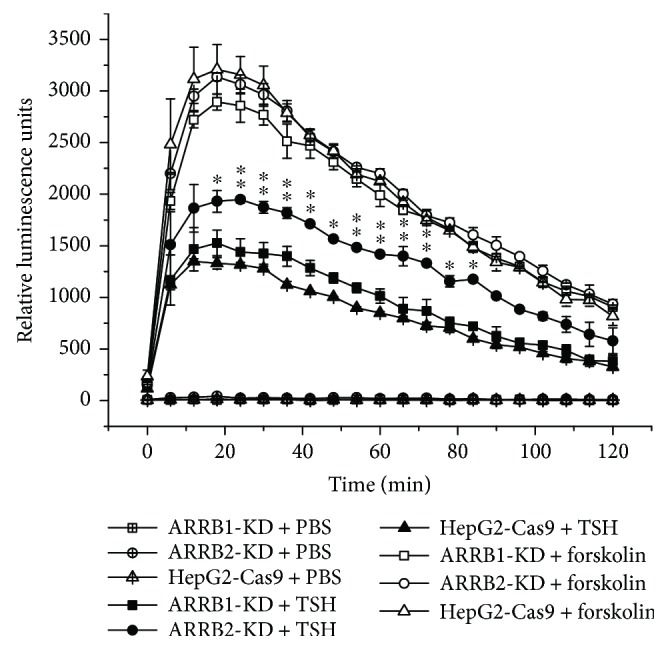
GloSensor analysis of cAMP level in ARRB1/2-knockdown HepG2 cells under TSH treatment. The cells were maintained in culture medium for 24 hours; then, the medium was changed with 100 *μ*L of CO_2_-independent medium with 2% *v*/*v* GloSensor cAMP Reagent (E1171, Promega) and 10% FBS for two hours. The cAMP signal was initiated by adding TSH, with forskolin (10 *μ*M) and PBS as positive and negative controls, respectively. GloSensor luciferase activities were measured on a microplate luminometer (Berthold Centro XS^3^ LB960) for 2 hours. For each treatment, 8 wells of cells were used as duplicated samples. For statistical analysis, ^∗^*p* < 0.05. Error bars are calculated as a standard error (SEM). ^∗∗^*p* < 0.01. Error bars are calculated as a standard error (SEM).

**Figure 5:**
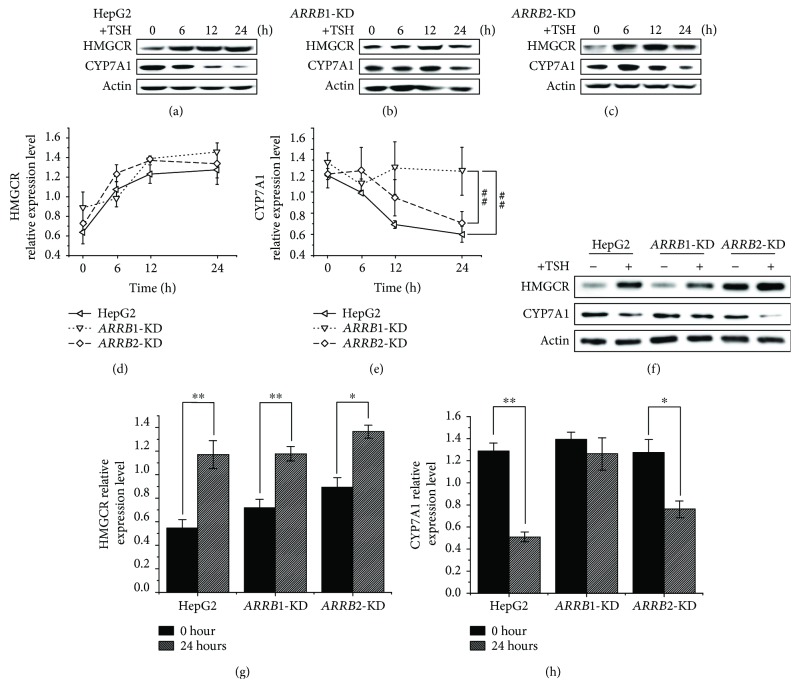
Immunoblotting analysis of cholesterol metabolism-related proteins in 24-hour TSH treatment. Cells were starved overnight and treated with 4 *μ*M TSH. Total protein was extracted at 0, 6, 12, and 24 hours after TSH was added. (a) HepG2-Cas9 cells, (b) *ARRB1*-knockdown HepG2-Cas9 cells, (c) *ARRB2*-knockdown HepG2 cells, and (d, e) grayscale analysis of relative expression level of HMGCR and CYP7A1 in TSH treatment of 0, 6, 12, and 24 hours. ^##^*p* < 0.01 indicated a statistical significance between genotypes. Error bars are calculated as a standard error (SEM). (f) Differences of HMGCR and CYP7A1 expression levels between ARRB1/2-knockdown and wild-type HepG2 cells with or without TSH treatment for 24 hours. (g, h) Grayscale analysis of HMGCR and CYP7A1 expression levels between ARRB1/2-knockdown and wild-type HepG2 cells treated with or without 24 h TSH treatment. ^∗^*p* < 0.05 indicated a statistical significance between time points within genotypes. ^∗∗^*p* < 0.01 indicated a statistical significance between time points within genotypes.

GPCRs are mainly involved in two general pathways, one through G protein activation and the other through ARRB-mediated pathways, which rely on the binding of ARRBs to the activated form of the GPCR. Previous studies have indicated that TSH is involved in liver cholesterol metabolism via the PKA/cAMP/CREB/HMGCR and SREBP2/HNF4*α*/CYP7A1 pathways [20, 23]; therefore, we next investigated whether ARRBs participated in this process, and if so, how. HepG2-Cas9 cells and *ARRB1/2* sgRNA-transfected HepG2-Cas9 cells were treated with 4 *μ*M TSH, and the GloSensor assay was performed to measure cAMP level in TSH treatment, along with forskolin and PBS treatment as positive and negative controls, respectively. The results are shown in Figure 4; during 2 hours of treatment, the cAMP levels of ARRB2-KD cells were obviously higher than those of ARRB1-KD cells and HepG2-Cas9 cells. ARRB2 deficiency increased the cAMP level, which was regarded as a convictive “readout” of the TSH-TSHR-PKA-cAMP effect. This result indicated that ARRB2 plays a more important role than ARRB1 in TSHR desensitization. For the analysis of the metabolic participants, protein levels were measured in long-time (24 h) TSH treatment [23]. ARRB1/2-KD cells along with HepG2-Cas9 cells were treated with 4 *μ*M TSH, and the protein levels of cholesterol metabolism participants were analyzed within 24 hours. The results are shown in Figure 5. In HepG2-Cas9 cells, TSH stimulation could upregulate the protein level of HMGCR, while the level of CYP7A1 was reduced (Figure 5(a)). These results were identical to those of our previous studies, which demonstrated that TSH can stimulate cholesterol synthesis and simultaneously inhibit cholesterol conversion [23]. In *ARRB1/2*-KD cells, the effect of TSH on HMGCR was not obviously influenced by ARRB deficiencies (Figures 5(b)–5(d) and 5(g)). However, the protein level of CYP7A1 tended to be higher in ARRB1-deficient cells than in *ARRB2*-KD and wild-type counterparts after 24 h TSH treatment (Figures 5(e), 5(f), and 5(h)). This result means that the inhibitory effects of TSH on CYP7A1 were weakened in *ARRB1*-KD cells but remained unchanged in *ARRB2*-KD, which indicated that ARRB1 may play more important roles in TSH regulation of cholesterol conversion.

### 3.4. Molecular Analysis of ARRB-Mediated Signaling Pathways Related to Cholesterol Metabolism during TSH Stimulation

**Figure 6:**
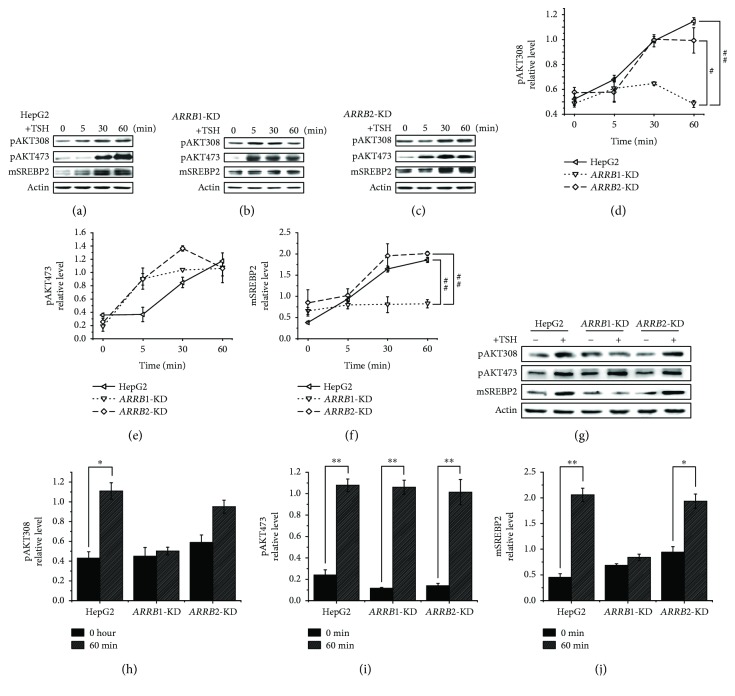
Expression analysis of *β*-arrestin-mediated signal pathways related to cholesterol metabolism in 60 min TSH treatment. Cells were starved overnight and treated with 4 *μ*M TSH. Total protein or nucleic protein was extracted at 0, 5, 30, and 60 min after TSH was added. Total protein was used for AKT phosphorylation level analysis, while nucleic component was used for the analysis of mature SREBP2, which is indicated as mSREBP2. (a) HepG2 cells, (b) ARRB1-knockdown HepG2-Cas9 cells, (c) ARRB2-knockdown HepG2 cells, and (d, e) grayscale analysis of relative phosphorylation level of AKT Thr308 and AKT Ser473 in TSH treatment of 0, 5, 30, and 60 min. (f) Grayscale analysis of relative expression level of mature SREBP2 in TSH treatment of 0, 5, 30, and 60 min. All experiments were repeated 3 times with similar results. ^#^*p* < 0.05 indicated a statistical significance between genotypes. ^##^*p* < 0.01 indicated a statistical significance between genotypes. (g) Differences of AKT phosphorylation and mature SREBP2 expression levels between ARRB1/2-knockdown and wild-type HepG2 cells treated with or without 60 min TSH treatment. (h, i, j) Grayscale analysis of AKT phosphorylation and mature SREBP2 expression levels between ARRB1/2-knockdown and wild-type HepG2 cells with or without TSH treatment for 60 min. ^∗^*p* < 0.05 indicated a statistical significance between time points within genotypes. ^∗∗^*p* < 0.01 indicated a statistical significance between time points within genotypes.

Previous studies indicated that TSH can influence the expression of liver CYP7A1 *in vivo* and *in vitro*. The nuclear receptor HNF4*α* mediates this process by binding to the promoter of CYP7A1, and TSH can inhibit HNF4*α* through the PI3K/Akt/SREBP2 signaling pathway [23]. The detailed relationship between TSH and AKT is not clearly understood. It was reported that besides acting as a negative regulator of GPCRs, ARRBs can also initiate a second wave of signal transmission independent of GPCRs through signaling pathways mediated by molecules such as protein kinase B (AKT); in this and several other metabolic pathways, ARRBs act as multifunctional adaptors [7]. Next, we determined whether ARRBs influence CYP7A1 through the AKT pathway. For signaling pathway analysis, protein levels and phosphorylation levels were measured in a short time after TSH treatment [21]. As shown in Figure 6(a), in HepG2-Cas9 cells, the phosphorylation levels of Thr308 and Ser473 of AKT were dramatically increased within 60 minutes of stimulation with 4 *μ*M TSH. In *ARRB1/2*-KD cells, AKT Ser473 phosphorylation levels were not dramatically different from those in HepG2-Cas9 cells (Figures 6(b), 6(c), 6(e), 6(g), and 6(i)), but the fluctuation of AKT Thr308 phosphorylation was reduced, especially in *ARRB1*-KD cells (Figures 6(b)–6(d), 6(g), and 6(h)). As a consequence, when stimulated with TSH, the increase in mature SREBP2 in HepG2-Cas9 cells showed decreased tendencies due to ARRB1 deficiency (Figures 6(b), 6(f), 6(g), and 6(j)). Since it was reported that the increase in mature SREBP2 could inhibit CYP7A1 expression, decreased fluctuation of mature SREBP2 in *ARRB1*-KD cells may be responsible for the heightened level of CYP7A1.

**Figure 7:**
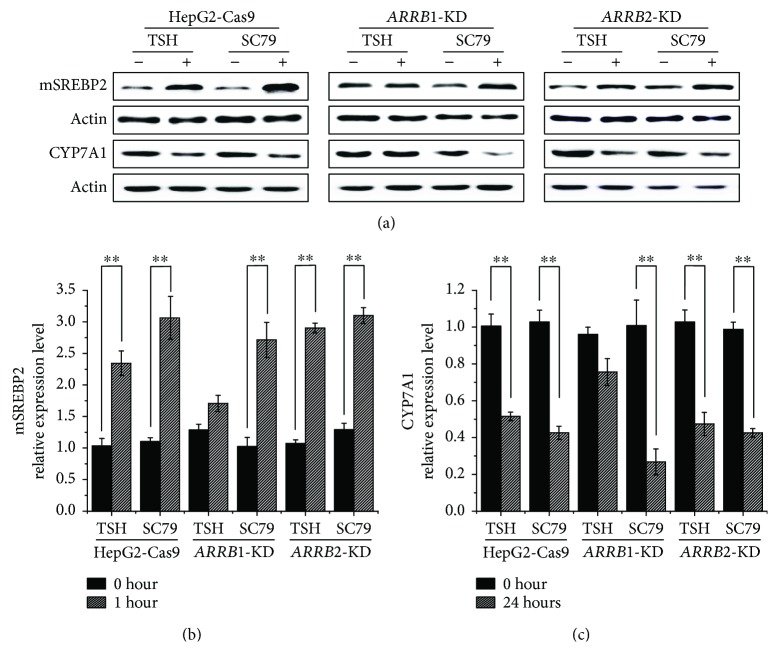
Immunoblotting analysis of cholesterol metabolism-related proteins and *β*-arrestin-mediated signal pathways in ARRB1/2-knockdown HepG2-Cas9 cells along with wild-type control. (a) Cells were starved and treated with TSH or AKT activator SC79 in a time course of either 1 hour or 24 hours. Protein extracts were collected and analyzed by Western blot with respective antibodies. The mature type of SREBP2 was indicated as mSREBP2. (b, c) Grayscale analysis of relative expression of mature SREBP2 and CYP7A1 was performed by ImageJ. All experiments were repeated 3 times with similar results. ^∗∗^*p* < 0.01. Error bars are calculated as a standard error (SEM).

Deficiencies in ARRB1 can lead to decreased phosphorylation of AKT Thr308 after TSH stimulation. To further verify the effect of AKT phosphorylation in the TSH-stimulated cholesterol conversion pathway, *ARRB1/2*-KD HepG2 cells along with HepG2-Cas9 cells were treated with the AKT activator SC79, which binds to the pleckstrin homology domain independent of GPCRs and ARRBs. SC79-bound AKT adopts a conformation favorable to phosphorylation by upstream protein kinases [24]. The levels of related molecules were analyzed by Western blot. The results are shown in Figure 7. After stimulation, protein samples were collected over a time course of 60 minutes for signaling molecule analysis and over 24 hours for cholesterol metabolism-associated protein analysis (Figure 7). The result turned out that compared to TSH, *ARRB1*-KD cells treated with SC79 showed an increased level of mature SREBP2 and decreased level of CYP7A1. It seemed that the inefficiencies of TSH in *ARRB1*-KD cells were complemented by SC79. These results indicated that AKT plays important roles in the regulation of SREBP2 and CYP7A1. Additionally, as an upstream stimulus of AKT, the function of TSH is at least partially dependent on ARRBs, especially ARRB1.

## 4. Discussion

Few previous studies have focused on the regulation of cholesterol metabolism by ARRBs. In Chinese hamster ovary (CHO) cells, the oxidative state of cell surface cholesterol is one of the factors involved in endothelin receptor type A (ETA) internalization, which occurs via caveolae or clathrin-coated pits [25]. Cholesterol was reported to participate in the endocytosis of lysophosphatidic acid-related receptors (LPA1) because the formation of LPA1-ARRBs is dependent on membrane cholesterol [26]. It seems that as an indispensable component of the cell membrane, cholesterol performs crucial roles in the interaction between GPCRs and ARRBs. ARRB2 was reported to be an important component of the mechanisms leading to cholesterol accumulation, which is a characteristic of epithelial cells associated with cystic fibrosis [27]. However, the role of ARRBs in cholesterol metabolism is rarely investigated.

In previous studies, TSHR was identified in liver tissues, and the major role of liver TSHR in cholesterol metabolism was demonstrated [20, 23]. In our present study, the effects of ARRB-mediated G protein-independent signaling on TSH-regulated cholesterol metabolism were reported. Deficiencies of ARRBs, especially ARRB1, led to a decreased cholesterol level in liver tissues and cells. The possible reason for this phenomenon might be related to a change in AKT phosphorylation level, which is shown to occur in the downstream of ARRB-related biased signaling pathways. Deficiencies in ARRBs led to a decreased level of TSH-stimulated AKT phosphorylation and a subsequent reduction in mature SREBP2. The AKT activator SC79, other than TSH, could upregulate AKT phosphorylation independent of TSHR and ARRBs, and SC79 treatment subsequently increased mature SREBP2 levels. Our results demonstrated that ARRBs, especially ARRB1, are involved in TSH-regulated cholesterol metabolism via the AKT pathway. This finding indicated that ARRBs may be further regarded as therapeutic targets in treating cholesterol disorders.

ARRBs were first discovered to function in GPCR desensitization [28]. As well-studied negative regulatory factors of GPCRs, ARRBs were mostly reported to have inhibitory effects on GPCR-related PKA pathways by both slowing the rate of cAMP production through GPCR desensitization and increasing the rate of cAMP degradation at the membrane [29, 30]. The trigger for ARRB-mediated GPCR desensitization and internalization is the stimulation of the GPCR ligand. In our study, the level of ARRB2 increased dramatically when cells were treated with TSH (Figures 1(b), 1(d), and 1(e)). This result indicated that ARRB2 might be involved in the first wave of TSHR-mediated cytological activity. Our results were consistent with the results of Frenzel et al.'s research, which demonstrated that ARRB2 showed more obvious colocalization with TSHR than ARRB1 under TSH treatment [11]. However, the protein levels of HMGCR, which can be considered downstream of the G protein-dependent pathway, increased dramatically in both ARRB-deficient cells and animals, even without extra TSH (Figure 3). This observation suggested that because ARRBs are negative regulators, the absence of ARRB1 or ARRB2 might reduce GPCR desensitization, leading to increases in downstream molecules. These losses of negative regulation may influence not only TSHR but also other GPCRs, not just the class A type. Together, our results partially confirmed that in liver tissues, ARRB2 might be involved in TSH-initiated cytologic activities and play more important roles than ARRB1 in desensitization of liver TSHR.

In addition to cholesterol synthesis, the cholesterol conversion process was taken into account to explain the influence of ARRBs on cholesterol. Conversion metabolism is important in maintaining cholesterol homeostasis. The key component, CYP7A1, is a rate-limiting enzyme in the synthesis of bile acids, which is a downstream metabolite of cholesterol. Our previous study illustrated that TSH-mediated cholesterol conversion was not dependent on PKA/cAMP but was dependent on PI3K/AKT/SREBP2, and CYP7A1/luc reporter activity was strongly inhibited by SREBP2 [23]. In our study, it seemed that ARRB2 showed a more obvious change in response to TSH stimulation. However, when ARRB2 was inactivated, the effects of TSH on CYP7A1 remained intact. Interestingly, ARRB1 deficiency caused an obvious variation in this process (Figure 5). Therefore, we speculated that the pathway of TSH regulating CYP7A1 might not depend on G protein signaling. Other than acting as negative regulators of GPCRs, ARRBs can initiate a second wave of cell signaling independent of G proteins [7]. This process, coupled with AKT signaling downstream of ARRBs, has multiple physiological effects, including carcinogenesis, cell apoptosis, motility, viability, and proliferation [8, 31–33]. It was reported that both ARRB1 and ARRB2 participated in the regulation of AKT phosphorylation level, in different styles. ARRB1 was most reported as the AKT phosphorylation activator during cell cycling, proliferation, and so on [34, 35], while ARRB2 showed a dephosphorylation effect by recruiting AKT1 and PP2A in GSK3b signaling [36, 37]. In our study, ARRB1 deactivation blunted the effect of TSH on AKT phosphorylation, which in turn downregulated the level of mature SREBP2 (Figure 6). These variations, which were due to ARRB1 deficiency, were complemented by the AKT activator SC79 (Figure 7). These results demonstrated that ARRB1 was necessary for TSH-related AKT signaling and is therefore involved in cholesterol conversion metabolism in liver tissues.
