# Corrigendum to “Beta-Arrestin 1 Mediates Liver Thyrotropin Regulation of Cholesterol Conversion Metabolism via the Akt-Dependent Pathway”

**DOI:** 10.1155/2018/3029602

**Published:** 2018-09-10

**Authors:** Shaona Niu, Hui Li, Wenbin Chen, Jiajun Zhao, Ling Gao, Tao Bo

**Affiliations:** ^1^Shandong Key Laboratory of Endocrinology and Lipid Metabolism, Institute of Endocrinology and Metabolism, Shandong Academy of Clinical Medicine, Shandong Provincial Hospital Affiliated to Shandong University, Jinan, Shandong 250021, China; ^2^Department of Endocrinology, Lin Yi People's Hospital Affiliated to Shandong University, Linyi, Shandong 276003, China; ^3^Medical College, Shandong University, Jinan, Shandong 250012, China; ^4^Scientific Center, Shandong Provincial Hospital Affiliated to Shandong University, Jinan, Shandong 250021, China; ^5^Department of Endocrinology and Metabolism, Shandong Provincial Hospital Affiliated to Shandong University, Jinan, Shandong 250021, China

In the article titled “Beta-Arrestin 1 Mediates Liver Thyrotropin Regulation of Cholesterol Conversion Metabolism via the Akt-Dependent Pathway” [1], the first affiliation was incomplete. The correct affiliation is shown above.
